# Degenerative Disorder of the Temporomandibular Joint Treated With Autologous Bone Marrow-Derived Stem Cells Using the Regentime Technique: A Case Report

**DOI:** 10.7759/cureus.34092

**Published:** 2023-01-23

**Authors:** Rita T Boulos, Cynthia F Najjoum, Elsa A El Asmar, Nassim H Abi Chahine

**Affiliations:** 1 Stem Cell Transplantation/Neurology, ACE Cells Lab UK Ltd., Sheffield Rotherham, GBR; 2 Stem Cell Transplantation/Infectious Diseases/Immunology, ACE Cells Lab UK Ltd., Sheffield Rotherham, GBR; 3 Stem Cell Transplantation/Functional Genomics/Proteomics, ACE Cells Lab UK Ltd., Sheffield Rotherham, GBR; 4 Stem Cell Transplantation/Neurological Surgery, ACE Cells Lab UK Ltd., Sheffield Rotherham, GBR

**Keywords:** tmj disorders, bone marrow-derived stem cells, autologous mononuclear stem cells, stem cell therapy, regentime therapy, temporomandibular joint degeneration

## Abstract

Temporomandibular joint (TMJ) disease is a type of degenerative musculoskeletal disorder that leads to morphological and functional abnormalities. It has a poorly understood progression with numerous independent and interrelated factors, which makes it difficult for the available treatment options to meet long-term demands.

We present the case of a 37-year-old woman who suffered from excruciating pain in the right temporomandibular joint, associated with limited mandibular movement. She was found to have imaging features of TMJ disorder. She underwent the Regentime procedure which uses autologous bone marrow-derived stem cells that are partially differentiated and redirected to the targeted tissue. Clinical follow-up showed total clinical recovery.

## Introduction

The temporomandibular joint (TMJ) and its related structures play an important role in controlling mandibular motion. They are essential for distributing pressures caused by everyday activities such as eating, swallowing, and speaking [1]. The TMJ is formed by the temporal bone glenoid fossa and the mandibular condyle [2]. It is a fibrocartilagenous joint with an articular disc and two synovial cavities allowing gliding and rotatory movements [3]. Intra-articular positional abnormality and excessive physical stress on the TMJ lead to dysfunctional joint remodeling, triggering TMJ degenerative changes [4].

Signs and symptoms of TMJ degeneration include clicking and crepitations during mandibular movement, pain over the joint, ear, and temporal fossa, mandibular movement limitation during opening, closure, protrusion, and lateral motion, headache, and tinnitus [5].

The condition affects most people in relatively moderate forms. Symptoms may improve considerably or disappear spontaneously over time. However, others may suffer from long-term severe pain. Physical therapy modalities, psychological therapies, and relaxation techniques can be used to treat TMJ disorders [6]. In terms of pharmaceutical agents, non-steroidal anti-inflammatory drugs (NSAIDs) are commonly prescribed, in addition to corticosteroids, hyaluronic acid, glucosamine, antidepressants, anticonvulsants, opioids, and muscle relaxants which may be transiently useful [7].

Meanwhile, the Regentime procedure attracts attention as an alternative approach toward joint tissue repair using autologous bone marrow-derived stem cells. Stem cells have become vital seed cells for tissue regeneration owing to their multilineage differentiation potential. We present the case of a 37-year-old patient diagnosed with TMJ degeneration in March 2019. She underwent stem cell therapy with the Regentime procedure in August 2020. Seven months after undergoing this therapy, the patient showed a major decrease in her symptoms.

## Case presentation

A 37-year-old woman with a history of multiple molar extractions during young adulthood was presented for chronic intractable pain in the right TMJ. She claimed a seven-year history of clicking sounds in her affected joint. She reported constant severe headaches, earaches, severe tension in the neck and shoulders, and throbbing facial pain for the past two years. She had difficulty maintaining daily activities such as chewing, swallowing, and speaking. She regularly consumed NSAIDs and tried intra-articular steroid injections, myorelaxants, opioids, anti-epileptics, physical therapy (such as deep tissue massage, dry needling, and thermotherapy), shiatsu, and different types of mouth guards, all of which failed to provide substantial relief. She reports an intractable increase in symptoms over the past year.

**Figure 1:**
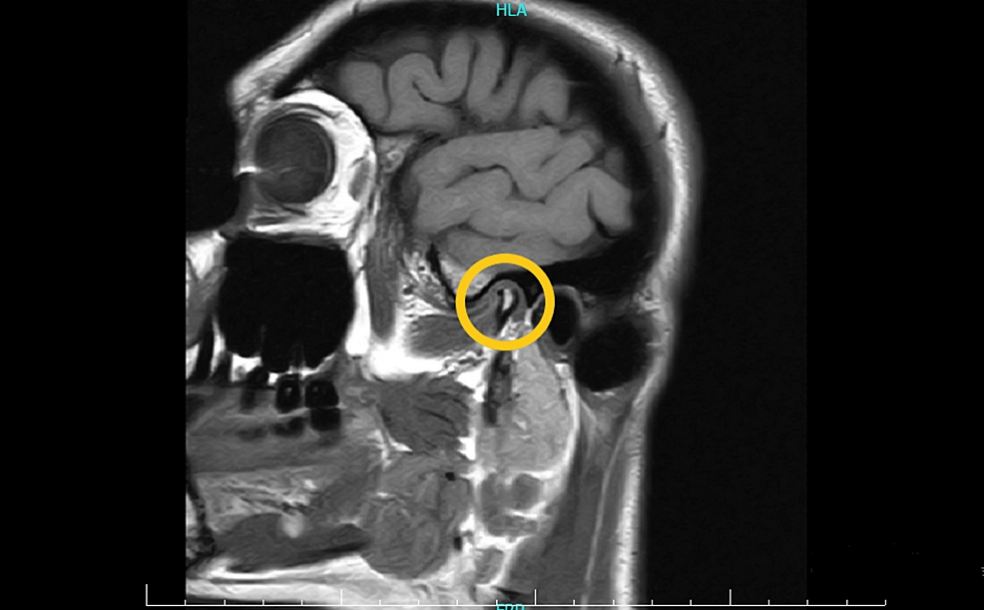
MRI of the patient's right temporomandibular joint before stem cell therapy MRI: Magnetic resonance imaging

Upon examination, her mouth opening was limited to 12 mm when measured between her maxillary and mandibular incisor incisal edges. The patient had painful and limited movements of the jaw. She showed swelling and tenderness on the right side of her face. Magnetic resonance imaging (MRI) was used to evaluate her right TMJ (Figure 1).

It showed anterior disc displacement with an irregular and crumbled appearance, along with condylar flattening and sclerosis. After proposing the Regentime procedure, the patient decided to attempt this therapy.

The Regentime procedure

The Pre-lab Stage

The patient was administered two doses of granulocyte colony-stimulating factor separated by 12 hours. A rise in her white blood cell count from 6,000/µL to 16,000/µL was noted 12 hours after the second dose.

The Bone Marrow Collection Stage

Bone marrow aspirate was obtained from both posterior superior iliac crests using heparinized aspiration syringes. The aspirate was then transferred to an empty blood bag under sterile conditions.

The Laboratory Stage

The bag was incubated for 12 hours on a slow three-dimensional laboratory shaker. Centrifugation was then performed, and the cellular buffy coat containing stem cells was extracted. This extract was incubated with cartilage ultra-filtrate (ACE Pico Cartilage, from ACE cells Lab Ltd. UK) and kept on a shaker for 12 hours.

The Transplantation Stage

After the second incubation, the patient received the final product as progenitor stem cells via direct TMJ injections: 1 ml in each of the superior and inferior synovial cavities. The patient received around 500 million stem cells per 1 ml.

The Post-transplantation Period

The patient experienced dull pain rated 8 to 9 on a numerical rating scale from 0 to 10 during the next 24 hours. She spent the night at the hospital for monitoring and was discharged the next morning. 

**Figure 2:**
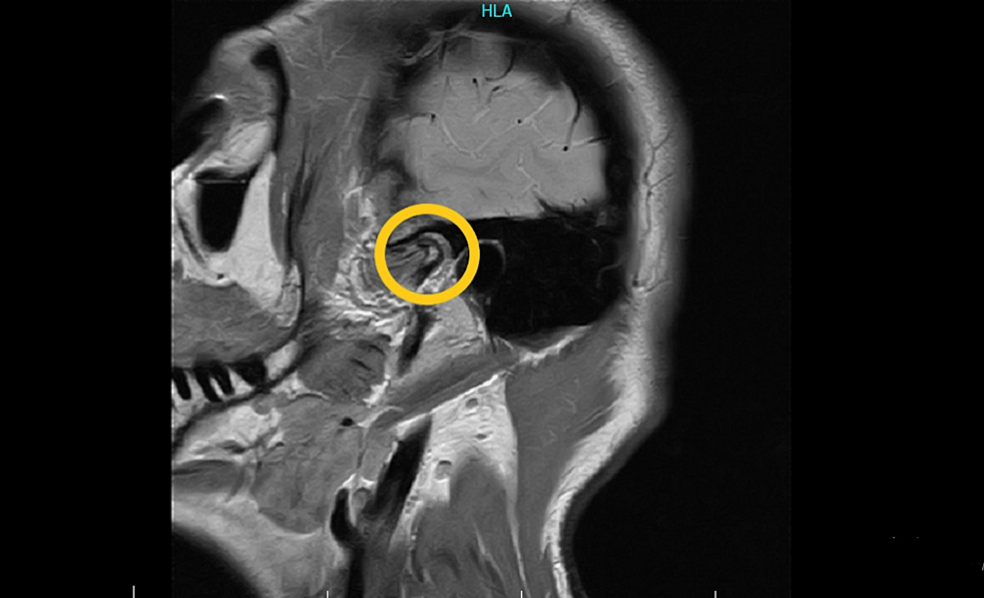
MRI of the patient's right temporomandibular joint one year after stem cell therapy MRI: Magnetic resonance imaging

Follow-up was carried out over two years after the Regentime procedure. Appointments on monthly basis first revealed minimal linear improvement of symptoms and a relative decrease in pain and medication use. At the seventh month post-Regentime therapy, the patient reported markedly rapid symptom relief and full regain of her TMJ functionality. She experienced no more pain during her daily activities. She regained full-range mandibular opening (around 60 mm) and reported painless chewing and swallowing. Despite her total clinical recovery, MRI done a year later showed no major changes in her right TMJ (Figure 2).

## Discussion

According to epidemiological studies, around 60-70% of the general population have TMJ disease signs, with one-quarter of them reporting symptoms and seeking treatment. The peak incidence is observed in the young population aged 20 to 40 years, with women being four times more likely to be affected [3]. Our patient is a young lady who has a history of multiple molar extractions which might have caused her internal derangement of the right TMJ. Several subsequent factors may have contributed to the dysfunctional remodeling of her TMJ leading to the progression of her case.

TMJ internal derangement is defined as the disc’s aberrant location with respect to the condyle and articular eminence [4]. According to the staging criteria for internal derangements of the TMJ, our patient had an intermediate to late-stage status: chronic pain, anterior disc displacement with degenerative remodeling, condylar flattening and sclerosis, chronic TMJ motion restriction (non-reducing disc acting as an obstacle to the gliding condyle upon mouth opening), and marked anatomical deformity with no disc perforation [8].

Internal derangement of the TMJ is a pathological condition related to TMJ osteoarthritis, which is particularly observed in cases with anterior disc displacement without reduction [9]. The association between TMJ anterior disc displacement and subsequent osteoarthritis is suggested to be causative [10]. In fact, degenerative osteoarthritic changes in the TMJ may develop as a result of mechanical and functional overloading that exceeds the joint normal adaptive capacity. Vascular endothelial growth factor (VEGF) secretion increases progressively in the overloaded articular cartilage and mediates destructive processes in osteoarthritis. VEGF upregulates matrix metalloproteinases (MMPs) and downregulates tissue inhibitors of matrix metalloproteinase resulting in an imbalance in the extracellular matrix components turnover toward increased degradation and subsequent cartilage destruction. VEGF also plays a role in the secretion of pro-inflammatory cytokines such as interleukin-1 (IL-1), interleukin-6 (IL-6), and tumor necrosis factor-α (TNF-α) which are involved in the progression of osteoarthritic changes [4,11].

Mesenchymal stem cells (MSCs) may be implicated in the treatment of immune-related and degenerative conditions such as TMJ osteoarthritis due to their broad immuno-modulatory ability and multidirectional differentiation potential. In fact, MSCs inhibit the production of MMP-13 and nuclear factor kappa-light-chain-enhancer of activated B cells (NF-κB), thereby downregulating IL-1ß production and suppressing joint inflammation. This further allows MSC-mediated chondrogenesis since NF-κB is thought to mediate IL-1ß and TNF-α inhibition of SOX9 expression in chondrocytes, an essential transcription factor involved in cartilage formation and the expression of chondrocyte-specific genes [12,13]. Similarly, other studies state that bone marrow-derived MSCs have chondrogenic differentiation potential allowing them to enhance the regenerative process of cartilage repair in TMJ osteoarthritis through the upregulation of key mediators involved in chondrogenesis such as SOX9, Col2, and aggrecan [14,15].

Furthermore, a randomized controlled clinical trial involving 30 patients with TMJ disease detected significant improvements after six and 12 months in the group of patients who received a bone marrow nucleated cell (BMNc) intra-articular injection compared with the group who received intra-articular hyaluronic acid. The BMNc group presented more important pain relief, higher chewing efficiency, and larger maximal interincisal opening compared with the second group. Similarly to our patient’s case, no evidence of cartilage repair was detected on MRI [16].

Due to the not fully understood pathophysiology of TMJ osteoarthritis and articular cartilage's low intrinsic regenerative capacity, in addition to the absence of an effective long-term treatment for symptomatic TMJ disease, an approach toward increasing TMJ healing capacity is largely needed. Bone marrow-derived mononuclear stem cell therapy presents therapeutic effects for people suffering from TMJ osteoarthritis, leading to a better quality of life. Despite the absence of MRI evidence of cartilage repair, our patient restored full functionality of her TMJ. She reported complete recovery of her symptoms seven months after treatment.

## Conclusions

TMJ disorder affects most people in relatively moderate forms; however, it can be debilitating to a small percentage of patients. We presented the case of a 37-year-old woman with intermediate/late-stage TMJ degeneration associated with chronic intractable pain who underwent stem cell therapy according to the Regentime procedure. Complete clinical recovery was reported seven months after treatment, in the absence of imaging evidence of cartilage repair. Large-scale clinical trials may be beneficial in demonstrating the therapeutic role of MSCs regarding TMJ disease.
